# Internal Oxidation of Silver-Based Alloys: Strengthening–Toughening Mechanisms and Microstructural Control

**DOI:** 10.3390/ma19214665

**Published:** 2026-11-02

**Authors:** Haicheng Zhu, Shaohong Liu, Limin Zhou, Han Mei, Hao Cui, Hongying Pei, Wenyan Zhou, Manmen Liu, Haigang Dong, Ming Wen, Li Chen, Wei Wang, Song Li, Jianjun Wang, Liang Zuo

**Affiliations:** 1 Key Laboratory for Anisotropy and Texture of Materials (Ministry of Education), School of Materials Science and Engineering, Northeastern University, Shenyang 110819, China; 2 State Key Laboratory of Precision Welding & Joining of Materials and Structures, Harbin 150001, China; 3 State Key Laboratory of Precious Metal Functional Materials, Yunnan Precious Metals Laboratory Co., Ltd., Kunming 650106, China; 4 AECC Shenyang Liming Aero-Engine Co., Ltd., Shenyang 110043, China; 5 Sino-Platinum Metals Semiconductor Materials (Yunnan) Co., Ltd., Kunming 650106, China

**Keywords:** silver-based alloys, internal oxidation, dispersion strengthening, electrical contact materials, microstructure control, strength–ductility synergy

## Abstract

Silver-based alloys are irreplaceable in high-reliability electrical contacts because silver has the highest electrical conductivity of all metals, yet its low strength and the intrinsic strength–ductility trade-off limit their use under increasingly severe service conditions. Internal oxidation—the selective in situ oxidation of reactive solutes in a chemically inert Ag matrix—converts these solutes into fine, dispersed oxides, thereby strengthening the alloy. This review critically assesses internal oxidation in silver-based alloys. The thermodynamic and kinetic criteria for selective oxidation are summarized, together with the Wagner diffusion-controlled framework describing the oxidation front. Using Ag-Mg and Ag-Sn as archetypal systems, we trace the microstructural evolution from coherent Mg-O nanoclusters and dispersed SnO_2_ particles to deleterious banded oxides, identifying band formation and under-oxidized cores as the two characteristic microstructural defects. The roles of temperature, time, oxygen partial pressure and third-element additions (Te, In, Sb, Bi, Ni) in widening the process window and refining the oxide dispersion are examined. Orowan bypassing, coherency strain and Hall–Petch grain refinement, and their coupling with microstructural characteristics, are discussed to provide guidelines for alloy design. Finally, outstanding questions and opportunities, including atomic-scale in situ characterization, predictive multiscale modeling and novel processing routes, are outlined.

## 1. Introduction

Silver-based alloys have long served as the backbone of high-performance electrical contact materials in defense, aerospace and high-reliability electronic devices [1]. Silver possesses the highest electrical conductivity of all metals (about 6.3 × 10^7^ S·m^−1^), an excellent thermal conductivity of ~429 W m^−1^ K^−1^ and a low, stable contact resistance, enabling reliable operation under high-frequency switching, high current density and harsh environments [2]. Copper-based conductors satisfy general engineering needs, but their performance stability under extreme or high-reliability conditions remains inadequate, leaving silver-based systems irreplaceable in such applications [3].

Different applications impose distinctly different property portfolios: signal-level contacts demand a low and stable contact resistance; load-bearing conductive components require high strength together with retained conductivity; and high-current switching contacts must additionally resist arc erosion and welding [4,5]. This diversity has produced a family of silver-based materials whose characteristics are compared in Table 1.

**Figure 1:**
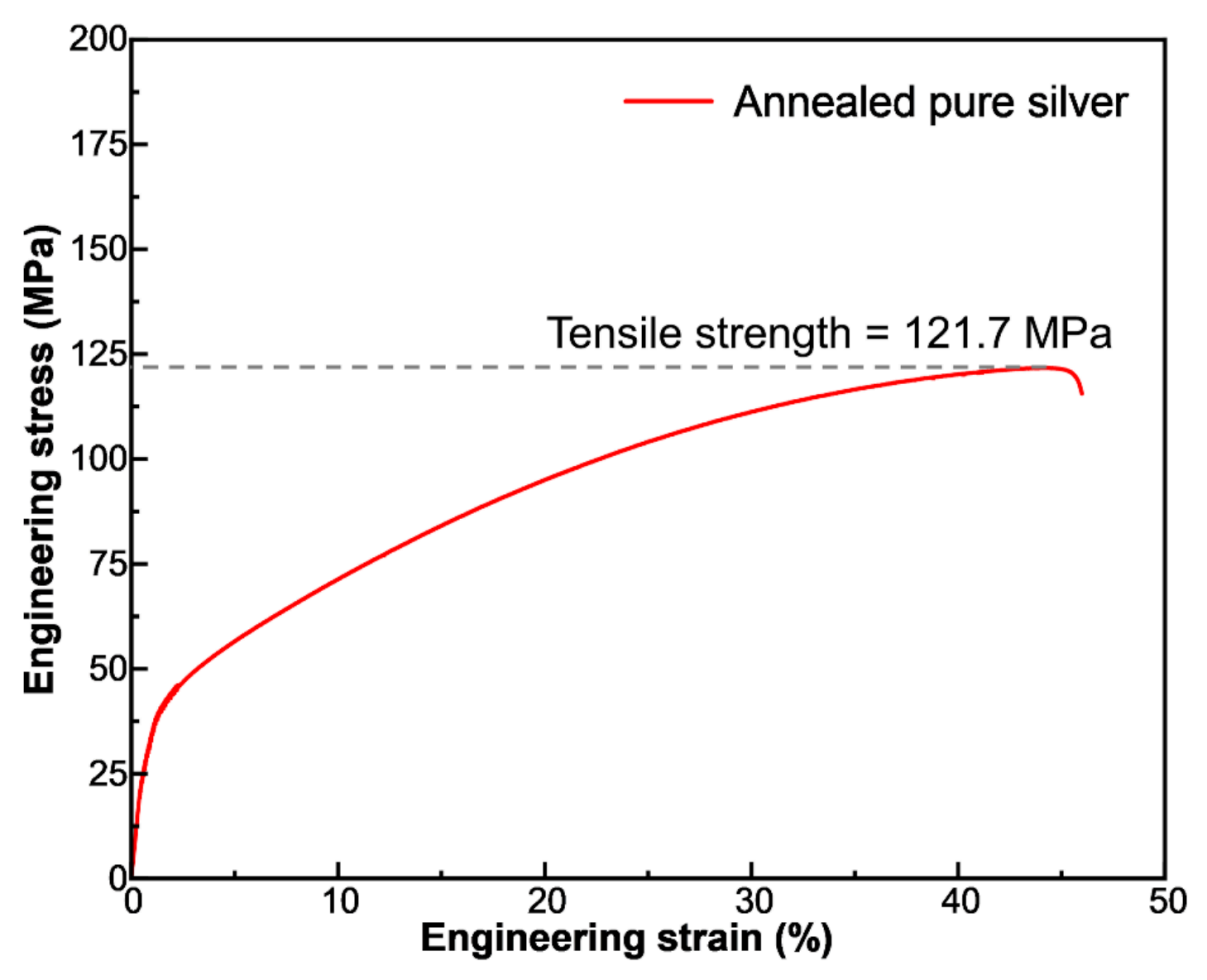
Stress–strain curve obtained at room temperature for a pure silver specimen [16].

Pure silver, despite its ductility, yields at only ~50 MPa at room temperature (Figure 1) and undergoes pronounced plastic flow under load, which precludes its use in high-load or high-reliability scenarios [16]. Conventional strengthening strategies—solid-solution strengthening, precipitation hardening, work hardening and second-phase strengthening—all improve mechanical properties to some extent, but the introduction of high densities of defects or poorly bonded second-phase interfaces may promote strain localization and premature fracture, thereby limiting ductility [17,18,19]. The core challenge for silver-based contact materials is therefore the simultaneous optimization of strength and ductility—a trade-off that conventional metallurgy resolves only poorly.

Internal oxidation offers a fundamentally different route. Dissolved oxygen diffuses through the Ag lattice and selectively oxidizes a reactive solute (e.g., Mg, Sn, Al, Zn), forming fine, dispersed oxide particles in situ within the Ag matrix. This process has two main advantages. First, the oxides impede dislocation motion and thereby provide substantial strengthening. Second, in situ formation may reduce the interfacial defects and weak bonding associated with externally introduced reinforcing particles, offering a promising route to an improved strength–ductility balance [20,21,22].

Although internal oxidation of silver-based alloys has been studied for decades, the formation mechanisms of the various internal oxide morphologies, and their influence on strengthening and embrittlement, remain to be clarified. Recent years have seen important progress in understanding the strengthening mechanisms of Mg-O nanoclusters, the formation and embrittlement mechanisms of oxide bands, and the effect of grain refinement on the strengthening–toughening behavior of internally oxidized alloys, providing new insight into the relationship between internal oxide microstructure and mechanical properties. Existing reviews on silver-based electrical contact materials have focused primarily on the fabrication processes, microstructural control and arc-erosion resistance of various material systems. However, for internal oxidation—an important route for tailoring the microstructure and properties of silver-based alloys—a consolidated analysis of the formation and evolution of internal oxides, together with the influence of these microstructural changes on mechanical properties and the underlying mechanisms, is still lacking.

This mini-review examines the formation and evolution of internal oxide microstructures in silver-based alloys, the mechanisms of strengthening and toughening, and the associated microstructural control strategies. Rather than aiming for exhaustive coverage, it draws on approximately 50 core primary studies identified through expert selection and supplemented by targeted searches of the recent literature, with emphasis on internal oxidation behavior, microstructural evolution and the resulting mechanical properties. On this basis, and comparing the Ag-Mg and Ag-Sn systems, we analyze the formation conditions of the different internal oxide structures and their effects on strengthening and embrittlement, establish links between processing, microstructure and mechanical properties, and provide guidance for the microstructural control and strengthening–toughening design of silver-based alloys.

## 2. Fundamentals of Internal Oxidation in Metals

Internal oxidation is a selective oxidation process that relies on both thermodynamic and kinetic conditions being met.

### 2.1. Thermodynamic and Kinetic Prerequisites for Selective Internal Oxidation

Thermodynamically, oxygen must preferentially react with the solute element B rather than the matrix element A [23,24]. The oxidation reactions of A and B can be expressed as

$$A+aO\to AO_{a}$$

$$B+bO\to BO_{b}$$

where $AO_{a}$ and $BO_{b}$ denote the oxides formed by the matrix and solute elements, respectively. Under identical temperature and oxygen availability, if the standard Gibbs free energy of formation of the solute oxide $BO_{b}$ is more negative than that of $AO_{a}$, element B has a stronger thermodynamic tendency toward oxidation and will be selectively oxidized. This condition is expressed as

$$\Delta G(AO_{a})>\Delta G(BO_{b})$$

where ΔG is the standard Gibbs free energy of formation of the respective oxides. Equivalently, the partial pressure of oxygen at the oxidation front, $P_{O_{2}}$, must satisfy

$${P_{O_{2}}^{A}>P}_{O_{2}}>P_{O_{2}}^{B}$$

where $P_{O_{2}}^{A}$ and $P_{O_{2}}^{B}$ are the critical oxygen partial pressures for the oxidation of element A and B, respectively. This ensures that oxygen reaching the internal oxidation front reacts selectively with the solute, while the matrix remains metallic.

Kinetically, the diffusion rate of oxygen within the matrix must be significantly higher than the outward diffusion rate of the solute atoms. The criterion for internal, rather than external, oxidation is [25]

$$D_{O}C_{O}^{S}\gg D_{B}C_{B}^{0}$$

where $D_{O}$ and $D_{B}$ are the diffusion coefficients of oxygen and solute B in the matrix, respectively, and $C_{O}^{S}$ and $C_{B}^{0}$ are their concentrations. When this inequality holds, oxygen penetrates the alloy much faster than solute atoms migrate to the surface, allowing a distinct internal oxidation front to be maintained.

### 2.2. Silver as the Ideal Internal-Oxidation Matrix

Silver exhibits several unique properties that make it an exceptionally suitable matrix for internal oxidation. Firstly, Ag_2_O is thermodynamically unstable above approximately 190 °C at atmospheric oxygen pressure and fully decomposes above 300 °C [26]. This means that within typical internal oxidation temperature ranges, the Ag matrix itself remains metallic, without forming an Ag oxide film on the surface that would impede oxygen diffusion into the alloy. Secondly, oxygen possesses remarkably high solubility and diffusivity in solid silver, often orders of magnitude greater than that of substitutional solute elements like Mg and Sn. For example, at elevated temperatures relevant to internal oxidation, the diffusion coefficients of Mg and Sn in silver are approximately 10^−14^–10^−13^ m^2^/s, while that of oxygen in silver is around 10^−10^–10^−9^ m^2^/s, a difference of several orders of magnitude [27,28,29]. The substantial difference in diffusion rates between oxygen and solute elements allows the kinetic selectivity condition for internal oxidation to be met over a wide range of solute concentrations, ensuring oxygen continuously penetrates the alloy without obstruction from surface oxide films, forming a deep and controllable internal oxidation front. Consequently, low-to-medium concentration silver-based alloys have become one of the most classic model systems for validating Wagner’s internal oxidation theory, with its theoretical predictions rigorously confirmed experimentally in these systems [30].

### 2.3. Wagner Kinetics and the Parabolic Rate Law

Wagner’s diffusion-controlled model forms the theoretical foundation for quantitatively describing internal oxidation kinetics, treating the process as a reaction diffusion problem. Oxygen diffuses into the alloy and reacts with the solute element at a continuously advancing reaction front, while solute atoms are assumed to be largely immobile. Under the assumptions of local thermodynamic equilibrium at the oxide–alloy interface, negligible solute back-diffusion, and complete consumption of solute at the reaction front, Wagner derived a parabolic relationship for the depth of the internal oxidation zone with time [24,25]:

$$x^{2}=kt$$

where *x* is the oxidation depth, *t* is the oxidation time and *k* is the internal oxidation rate constant. The constant k depends on oxidation temperature, oxygen partial pressure and solute concentration, and can be further expressed as [24,25]

$$k=\frac{{2C}_{O}^{S}}{bC_{B}^{0}}D_{O}$$

where $C_{B}^{0}$ is the initial mole fraction of the solute in the alloy, b is the stoichiometric coefficient of oxygen in the oxide $BO_{b}$, $D_{O}$ is the diffusion coefficient of oxygen in the matrix and $C_{O}^{S}$ is the mole fraction of oxygen at the alloy surface.

This formula yields three important conclusions:

1. Depth time kinetics: The internal oxidation depth is proportional to the square root of time:

$$x\propto t^{1/2}$$

Consequently, the advance rate of the oxidation front decreases with time:

$$\frac{dx}{dt}\propto t^{-1/2}$$

As material thickness increases, the time required for complete internal oxidation across the entire cross-section significantly lengthens. For example, doubling the material thickness quadruples the required oxidation time. This kinetic aspect explains why bulk electrical contact materials and clad materials often exhibit incomplete oxidation in their central regions, resulting in a coexistence of a harder outer layer and a softer interior.

2. Solute-concentration dependence: According to the Wagner relationship,

$$x^{2}\propto \frac{1}{C_{B}^{0}}$$

As the solute content increases, more solute atoms need to be oxidized per unit volume, and oxygen entering the matrix is consumed more rapidly, reducing the advance rate of the internal oxidation front. More critically, as the oxidation front slows down, solute atoms spend more time at the front, providing ample opportunity for significant coarsening, lateral aggregation and preferential alignment of oxides along grain boundaries. This is a kinetic reason for the formation of banded oxide structures at high solute contents [31].

3. Influence of oxygen partial pressure and temperature: When solute content, oxidation time and oxide stoichiometry remain constant, the change in internal oxidation depth can be approximately expressed as

$$x^{2}\propto C_{O}^{S}D_{O}$$

The equilibrium oxygen concentration at the alloy surface, $C_{O}^{S}$, is primarily influenced by the environmental oxygen partial pressure, while the oxygen diffusion coefficient in the silver matrix, $D_{O}$, is mainly controlled by temperature. Thus, oxygen partial pressure and temperature jointly regulate the advance of the internal oxidation front by altering surface oxygen concentration and oxygen diffusion capability. Increasing oxygen partial pressure enhances the solubility of oxygen at the alloy surface, increasing the flux of oxygen diffusing into the alloy and thus accelerating the internal oxidation rate, leading to a deeper internal oxidation layer in the same timeframe [32]. Higher oxygen partial pressure can also increase the number of oxide nuclei, promoting simultaneous oxidation at more sites and favoring the formation of smaller, more dispersed internal oxidation products [33]. Conversely, at lower oxygen partial pressures, insufficient oxygen supply may limit the advance of the internal oxidation front. Temperature primarily accelerates the internal oxidation front by affecting the oxygen diffusion coefficient in the silver matrix. However, temperature also influences solute diffusion, oxide nucleation and grain growth. Therefore, excessively high temperatures can also promote oxide coarsening, enrichment at grain boundaries or the formation of banded structures, thereby degrading the uniformity of the internal oxidation microstructure.

## 3. Microstructural Evolution in Internally Oxidized Ag-Mg and Ag-Sn Systems

### 3.1. The Ag-Mg System: Coherent Nanocluster Strengthening and Band Formation

The Ag-Mg alloy system is a classical model for studying internal oxidation strengthening behavior. Numerous studies have shown that internal oxidation of this system in situ forms nanoscale Mg-O clusters within the matrix [34,35,36,37]. These Mg-O clusters maintain a coherent or semi-coherent relationship with the Ag matrix, causing significant lattice distortion, which substantially increases the alloy’s strength and hardness [38,39,40].

**Figure 2:**
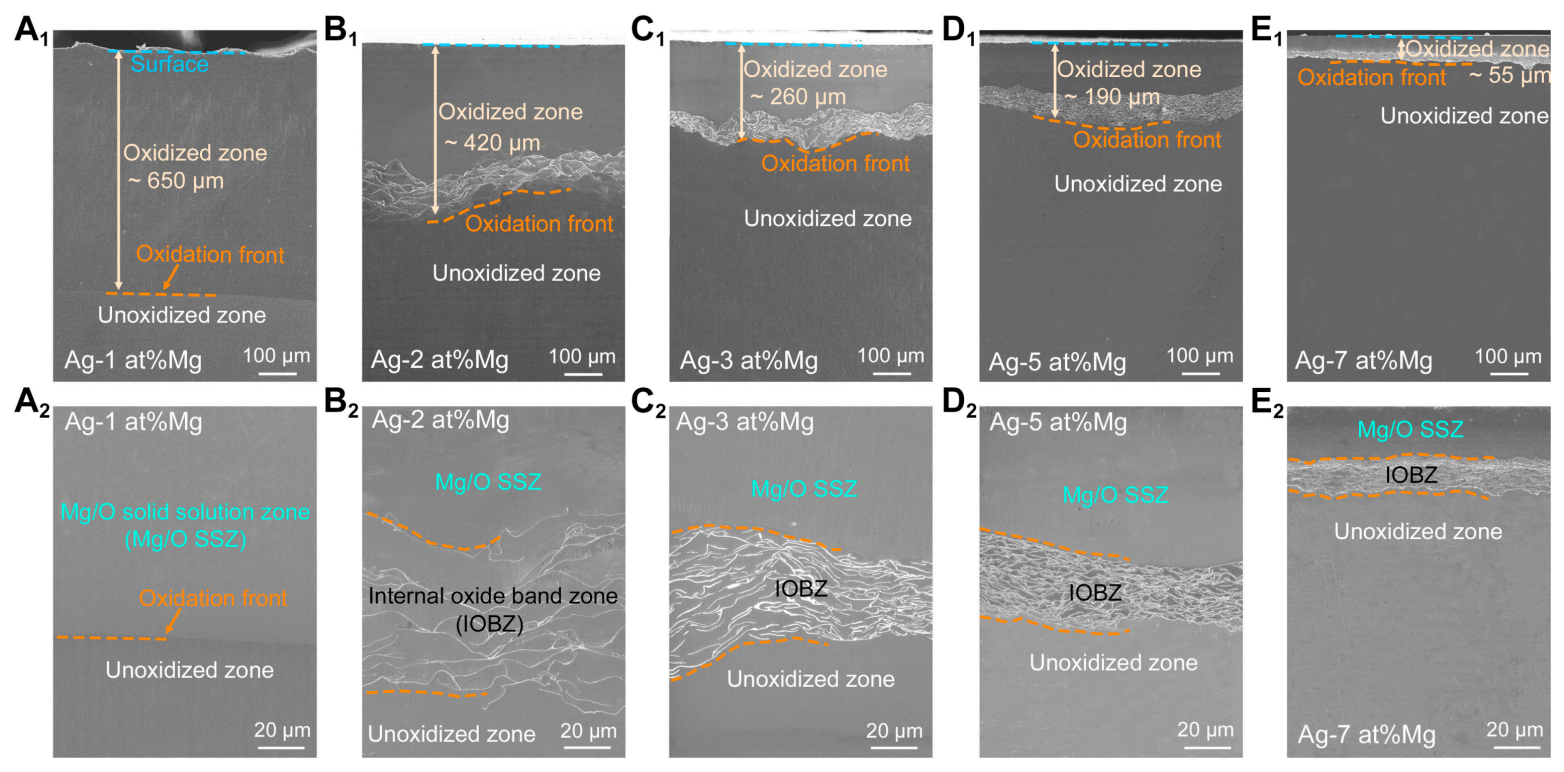
Typical microstructures of the Mg-O solid-solution zone, internal oxide band zone, and unoxidized zone in Ag-Mg alloys with different Mg contents [41]. (**A_1_**) Ag-1 at.% Mg, overview; (**A_2_**) enlarged Mg-O solid-solution zone (SSZ) and oxidation front; (**B_1_**) Ag-2 at.% Mg, overview; (**B_2_**) enlarged SSZ and internal oxide band zone (IOBZ); (**C_1_**) Ag-3 at.% Mg, overview; (**C_2_**) enlarged SSZ and IOBZ; (**D_1_**) Ag-5 at.% Mg, overview; (**D_2_**) enlarged SSZ and IOBZ; (**E_1_**) Ag-7 at.% Mg, overview; (**E_2_**) enlarged SSZ and IOBZ.

Zhu et al. prepared Ag-Mg alloys with Mg contents of 1, 2, 3, 5 and 7 at.% by melting, and subjected them to internal oxidation at 800 °C in pure oxygen for 2 h. After internal oxidation, high-Mg-content Ag-Mg alloys typically exhibit three distinct microstructural zones across their cross-section: a Mg-O solid-solution zone near the surface (initially often referred to as a precipitate-free zone, but later confirmed to contain approximately 3 nm Mg-O clusters coherent with the Ag matrix, difficult to observe with conventional SEM); an internal oxide band zone between the Mg-O solid-solution zone and the oxidation front, characterized by numerous band-like MgO precipitates, generally parallel to the surface; and an unoxidized zone in the central region of the alloy (Figure 2) [41].

**Figure 3:**
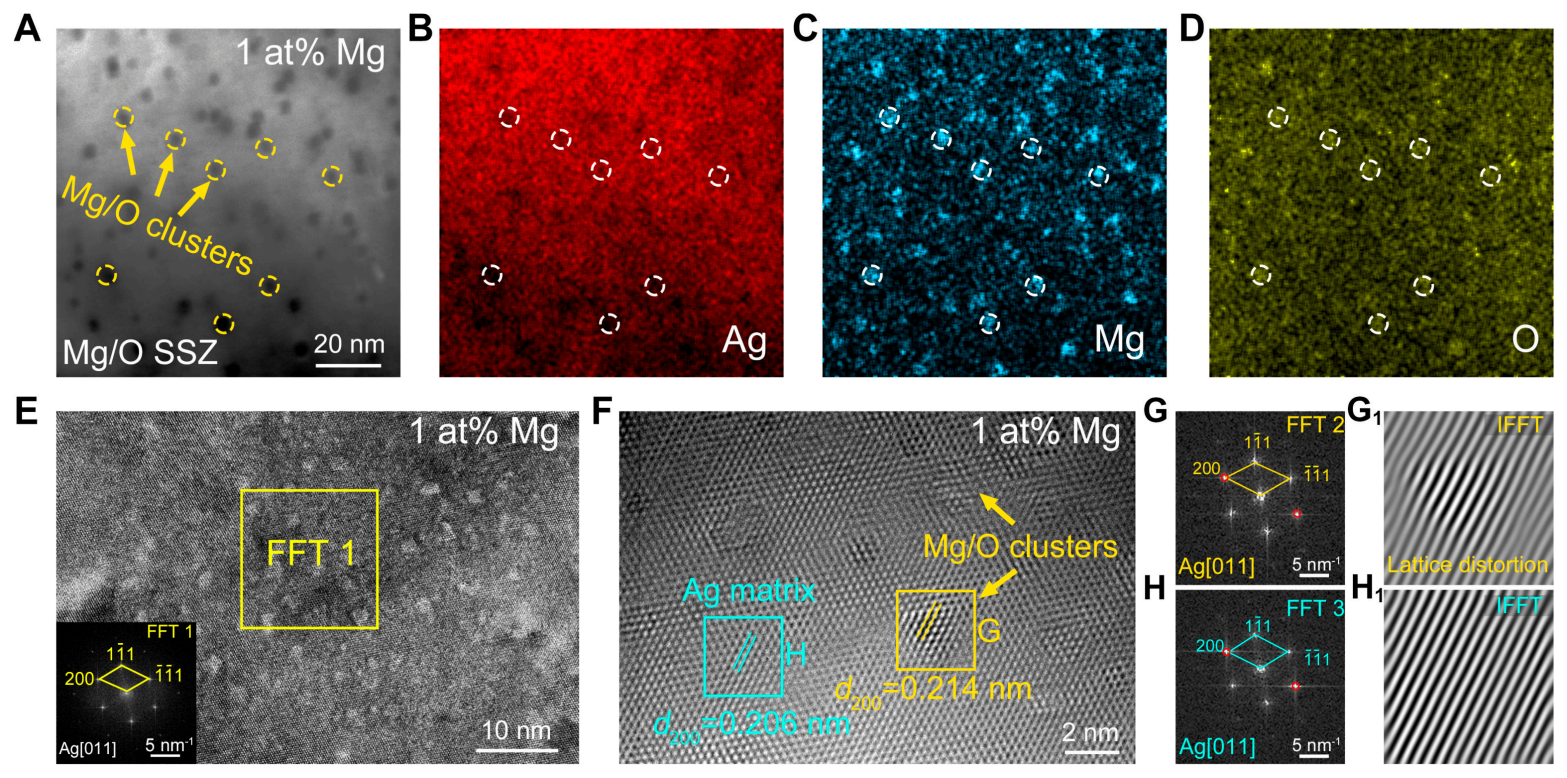
Nanoscale Mg-O clusters uniformly dispersed in the Ag matrix [41]. (**A**) HAADF-STEM image; (**B**) Ag, (**C**) Mg, and (**D**) O elemental maps; (**E**) HRTEM image with the corresponding FFT pattern; (**F**) HRTEM image showing coherent Mg-O clusters; (**G**) FFT image of the cluster-containing region; (**H**) FFT image of the Ag matrix; (**G_1_**) IFFT images of the cluster-containing region; (**H_1_**) IFFT images of the Ag matrix.

Figure 3 shows typical coherent Mg-O nanoclusters, approximately 3 nm in size, within the Mg-O solid-solution zone of the Ag matrix [41]. Douglass et al. observed dispersed clusters with a particle size of about 5 nm in internally oxidized Ag-Mg alloys, resulting in a microhardness of up to 300 VHN, about 5 times that of pure silver [42]. Goretta et al. showed that adding a small amount of Mg can significantly enhance the strength of internally oxidized Ag-Mg alloys. After internal oxidation, the yield strength of Ag-1.2 at.% Mg alloy increased dramatically from approximately 48 MPa in the unoxidized state to about 383 MPa [43]. Zhu et al. found that as the Mg content increased from 1 at.% to 7 at.%, the surface hardness of the alloy increased from 178 HV to 224 HV, mainly attributed to the increased density of in situ precipitated Mg-O clusters with increasing Mg content, leading to more significant matrix strengthening [41].

The rapid diffusion of oxygen and rapid depletion of Mg stabilize the cluster size and prevent coarsening. Charrin et al. studied Ag-Mg alloys with 0.04 at.% and 0.35 at.% Mg internally oxidized at 803 K, finding that the high reaction rate between oxygen and Mg allowed oxygen to be quickly fixed upon reaching the reaction zone, forming approximately 1 nm Mg-O clusters [44,45,46]. During subsequent internal oxidation, the lack of continuous Mg supply inhibited Ostwald ripening, and the Mg-O clusters maintained their initial size [47,48]. The fine and stable Mg-O clusters provide a microstructural basis for effective strengthening of Ag-Mg alloys.

**Figure 4:**
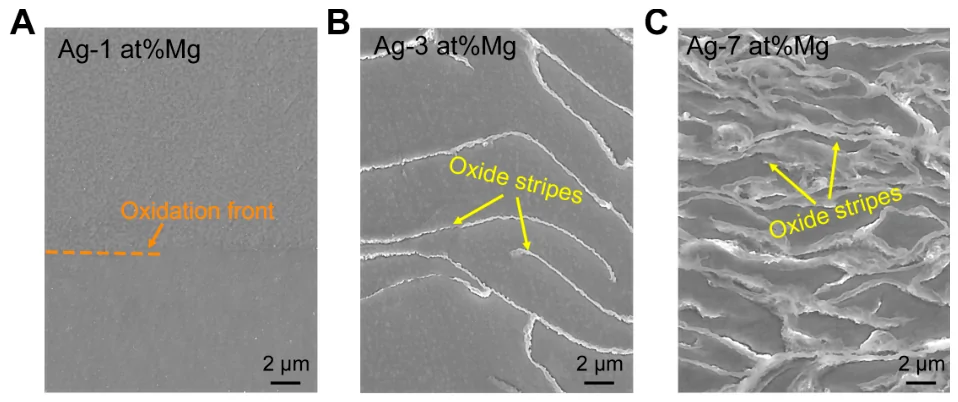
Evolution of oxide morphology in Ag-Mg alloys with increasing Mg content, showing the transition from uniform dispersion to stripe-like oxide structures [41]. (**A**) Ag-1 at.% Mg without oxide stripes; (**B**) Ag-3 at.% Mg with oxide stripes; (**C**) Ag-7 at.% Mg with extensive oxide stripes.

Mg content is a critical factor in controlling the microstructure of internally oxidized Ag-Mg alloys. In low-Mg-content Ag-Mg alloys, the concentration of Mg atoms per unit volume is low, requiring less oxygen for the internal oxidation reaction. A large amount of unreacted oxygen rapidly diffuses into the alloy, forming low-density Mg-O cluster regions and a deep internal oxidation depth. As Mg content increases, the density of Mg-O clusters in the oxidized region increases, and the oxidation front advance rate decreases. Unoxidized Mg then has sufficient time to accumulate at the oxidation front, promoting the growth of Mg-O clusters into larger particles, eventually evolving into MgO internal oxide bands. Zhu et al. showed that for Ag-Mg alloys internally oxidized at 800 °C, when Mg content was 1 at.%, the internal oxidation microstructure primarily consisted of Mg-O nanoclusters; when Mg content increased to 2 at.% or higher, band-like structures composed of nanocrystalline MgO gradually appeared near the oxidation front (Figure 4) [41]. Charrin et al. reported that MgO bands formed when Mg content exceeded 3 at.% during internal oxidation at 400–750 °C, while Ag-1.32 at.% Mg alloy did not form MgO bands [49]. This discrepancy highlights the sensitivity of band formation to specific processing parameters and warrants further investigation.

### 3.2. The Ag-Sn System: SnO_2_ Dispersion and the Banding Problem

Among Cd-free electrical contact materials, the Ag-Sn system is one of the most representative [50,51]. Good arc erosion resistance and anti-welding performance generally require a high oxide volume fraction [52]. Yet, the microstructural evolution of this system is subject to a well-known trade-off: a higher Sn content is desirable in terms of service performance, but it also favors the formation of deleterious banded oxides [53]. Previous studies prepared Ag-Sn alloys with Sn contents of 3.0–8.1 at.% by melting and rolling, and internally oxidized them at 600–800 °C to examine how Sn content governs microstructural evolution [30,54].

At low Sn contents, internal oxidation produces fine, equiaxed SnO_2_ particles that are uniformly dispersed in the silver matrix. Because these particles remain discrete rather than interconnected, oxygen can permeate the oxidized zone and the oxidation front continues to advance [55]. Such fine dispersions provide effective dispersion strengthening and markedly improve the arc erosion resistance and anti-welding performance [56]. In Ag-3 at.% Sn alloys oxidized in air and in high-pressure oxygen, a thick, uniform oxidation layer forms, with SnO_2_ particles of about 200 nm distributed throughout the oxidized zone; the oxidation depth scales linearly with the square root of time, consistent with Wagner’s model [30].

As Sn content increases, the concentration of Sn per unit volume rises, and oxygen is consumed more rapidly during diffusion, leading to a decrease in the oxidation front advance rate. Additionally, noticeable differences in the oxidation front advance rate emerge at various locations. Lee et al. showed that the oxidation front of Ag-3.5 at.% Sn binary alloy exhibited a jagged morphology, indicating variations in local oxidation rates and the onset of passivation or hindrance [54]. As the oxidation front slows, Sn atoms from the unoxidized region have sufficient time to accumulate at the front, leading to coarsening of SnO_2_ particles in the oxidation front region.

When Sn content exceeds approximately 5 at.%, oxygen is rapidly consumed as it diffuses inward, leading to the formation of high-density SnO_2_ particles in the internal oxidation zone [57,58]. These particles hinder the diffusion of oxygen into the unoxidized region, resulting in an unoxidized core. Due to the very slow advance of the oxidation front, a dense, continuous SnO_2_ oxide band forms at the oxidation front. These continuous SnO_2_ oxide bands act as physical barriers to oxygen diffusion, further impeding oxygen penetration and ultimately halting sustained internal oxidation, promoting a transition from internal oxidation to external oxidation. For instance, in Ag-8.1 at.% Sn alloy, internal oxidation is primarily confined to the surface layer, while the interior remains in an unoxidized state [54].

### 3.3. Formation of Banded Oxide Structures: Mechanisms and Consequences

Ag-Mg and Ag-Sn alloys exhibit similar patterns of internal oxidation microstructural evolution, governed by the interplay of inward oxygen diffusion, solute diffusion and oxidation reactions. In Ag-Mg alloys, the formation of MgO bands results from a progressive slowdown of the oxidation front, which allows Mg from the unoxidized interior to accumulate at the front. During internal oxidation, oxygen diffuses inward from the surface and reacts with the solute. At high solute contents, oxygen is continuously consumed as it advances, decelerating the oxidation front. Simultaneously, solute atoms from the unoxidized region migrate toward and accumulate at the front, promoting extensive nucleation and growth of oxide particles that eventually interconnect into continuous or semi-continuous band structures roughly parallel to the surface. As these bands develop, they increasingly impede further inward oxygen diffusion, reinforcing the tendency for continued band formation.

A structurally analogous evolution occurs in Ag-Sn alloys. At low Sn contents, oxygen diffuses into the Ag matrix and reacts with Sn to form fine, uniformly dispersed SnO_2_ particles. As the Sn content increases, greater oxygen consumption decelerates the oxidation front, Sn atoms accumulate near the front, and SnO_2_ particles nucleate densely and coarsen. These oxide-rich regions further impede inward oxygen diffusion and may eventually develop into banded structures. Consequently, when the Mg content reaches or exceeds 2 at.%, or the Sn content exceeds 5 at.%, internal oxide band structures can be observed under appropriate internal oxidation conditions [41,49,53]. Banded oxide structures are detrimental to the strength and ductility of the material [59].

**Figure 5:**
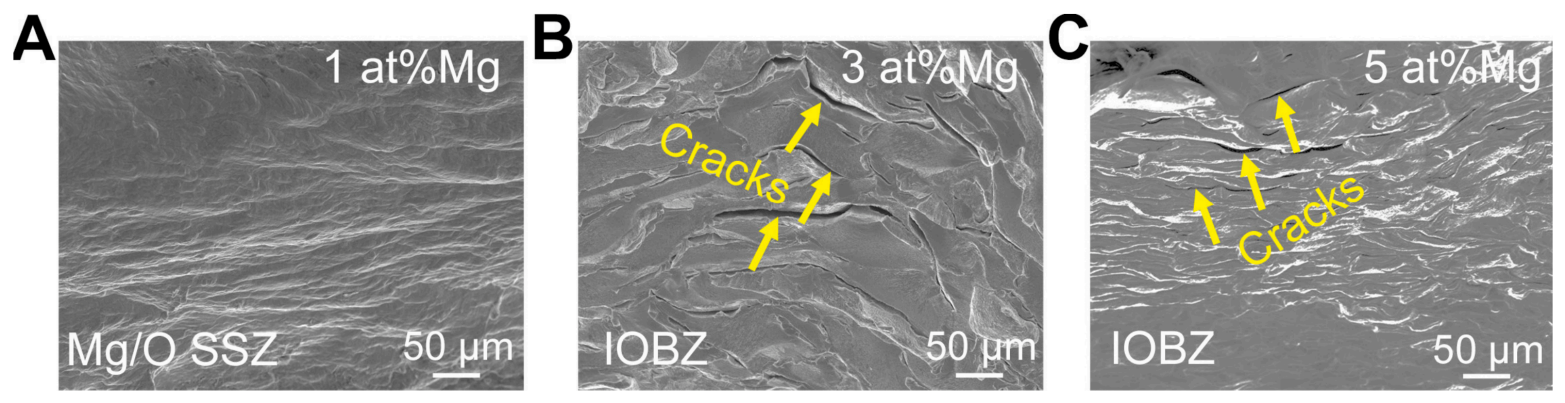
Typical fracture surfaces of internally oxidized Ag-Mg alloys with different Mg contents [41]. (**A**) Ag-1 at.% Mg, showing the Mg-O SSZ; (**B**) Ag-3 at.% Mg, showing cracks in the IOBZ; (**C**) Ag-5 at.% Mg, showing cracks in the IOBZ.

The presence of internal oxide bands progressively leads to reduced ductility and even brittle fracture in Ag-Mg alloys [42]. Goldacker et al. showed that when Mg content was below approximately 3 at.%, with no or few internal oxide bands, internally oxidized Ag-Mg alloys retained significant subsequent plastic workability [49,60,61]. However, with further increases in Mg content, alloy ductility decreased significantly. Zhu et al. further demonstrated that Ag-Mg alloys with 1 at.% Mg, exhibiting no internal oxide bands, showed ductile fracture characteristics, whereas alloys with 2 at.% Mg or higher exhibited distinct brittle fracture. Coarse MgO bands with poor interface matching with the matrix easily induce stress concentrations under load, acting as crack initiation sites and leading to material embrittlement (Figure 5) [41].

**Figure 6:**
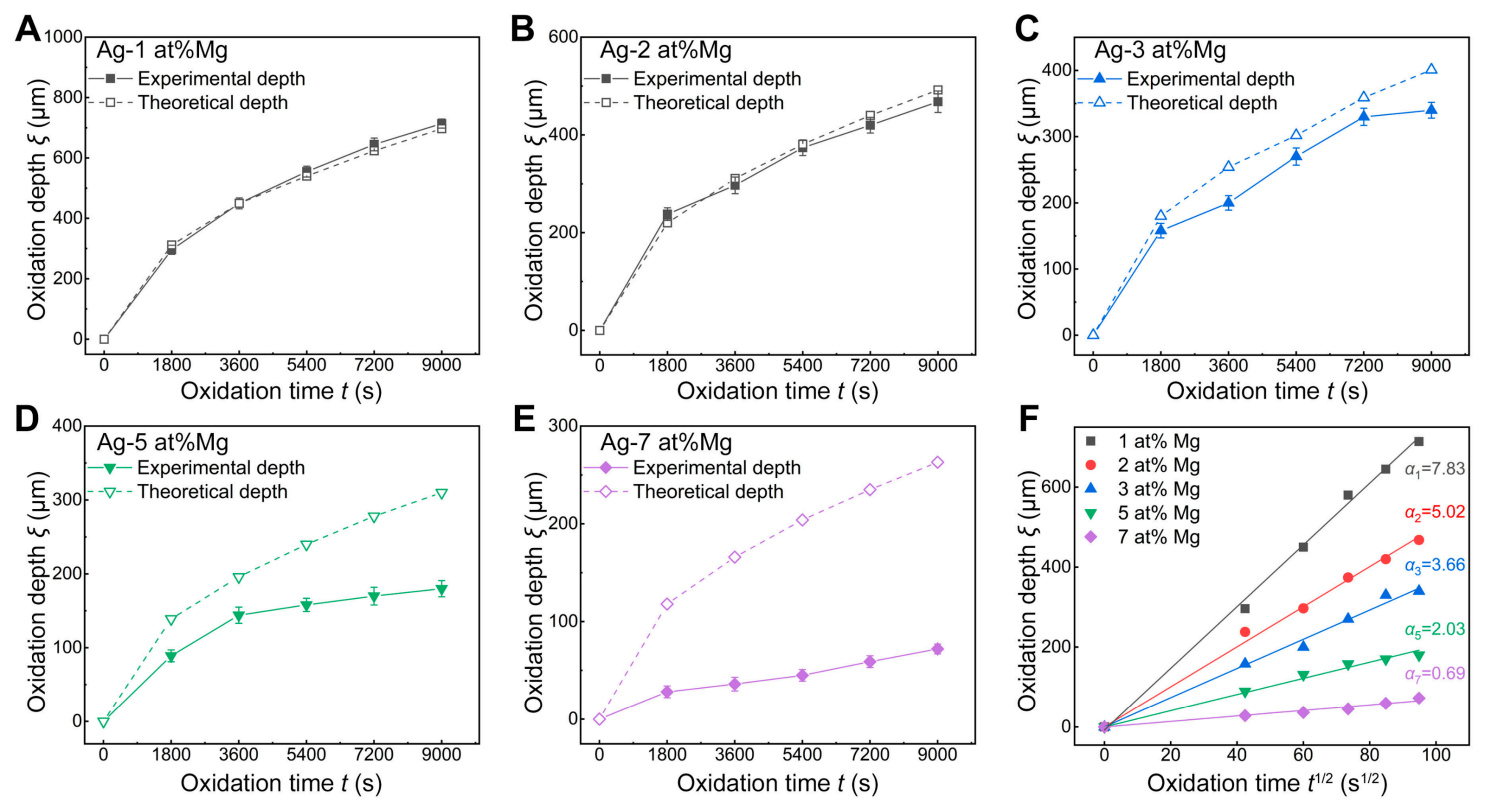
Internal oxidation kinetics at different Mg contents, showing that Mg content significantly affects the evolution of oxidation depth [41]. Comparison of experimental and theoretical oxidation depths for (**A**) 1; (**B**) 2; (**C**) 3; (**D**) 5; (**E**) 7 at.% Mg; (**F**) experimental oxidation depth versus the square root of oxidation time.

Furthermore, the formation of internal oxide bands significantly reduces the internal oxidation rate. Figure 6A–E show the experimental and theoretical internal oxidation depths for Ag-Mg alloys containing 1, 2, 3, 5 and 7 at.% Mg at various oxidation times. For all compositions, the internal oxidation depth increases progressively with time but at a diminishing rate. At low Mg contents, the experimental values agree closely with the theoretical predictions; as the Mg content increases, the two diverge noticeably, with the experimental oxidation depth falling well below the theoretical value in high-Mg alloys that develop internal oxide bands. Figure 6F summarizes these results, showing an approximately linear relationship between internal oxidation depth and the square root of oxidation time for all compositions. As the Mg content increased from 1 to 7 at.%, the fitted slope of oxidation depth versus the square root of time decreased from approximately 7.83 to 0.69 μm·s^−1/2^ [41]. This phenomenon, where internal oxide bands reduce the internal oxidation rate, is even more pronounced in Ag-Sn alloys. Studies have shown that when Sn content is 5 at.%, the corresponding fitted slope of Ag-Sn alloy at 800 °C can drop below 0.6 μm·s^−1/2^ [54]. Further increasing the Sn content can promote the formation of continuous SnO_2_ oxide bands, which hinder oxygen penetration and may eventually lead to a transition from internal to external oxidation.

## 4. Process-Parameter and Compositional Control of Microstructure

### 4.1. Oxidation Temperature: The Primary Control Lever

Temperature is a crucial process parameter influencing the internal oxidation behavior of silver-based alloys. Since internal oxidation is typically diffusion controlled, increasing temperature significantly enhances oxygen diffusion in the silver matrix, accelerating the inward advance of the internal oxidation front [23,62]. However, the effect of temperature on internal oxidation is not limited to an increased oxidation rate. Processes such as solute atom migration, oxide coarsening and matrix grain growth also accelerate with increasing temperature [63]. Therefore, temperature not only determines the depth of the internal oxidation layer but also significantly alters the size and distribution of internal oxides and the cross-sectional microstructure of the alloy, making it a critical parameter for controlling the internal oxidation microstructure of silver-based alloys. Higher temperatures generally promote faster oxidation but can also lead to coarser oxides and greater non-uniformity [64,65]. Thus, an optimal temperature range often exists to balance oxidation kinetics with microstructural refinement.

### 4.2. Oxidation Time: Kinetic Control via the Parabolic Law

**Figure 7:**
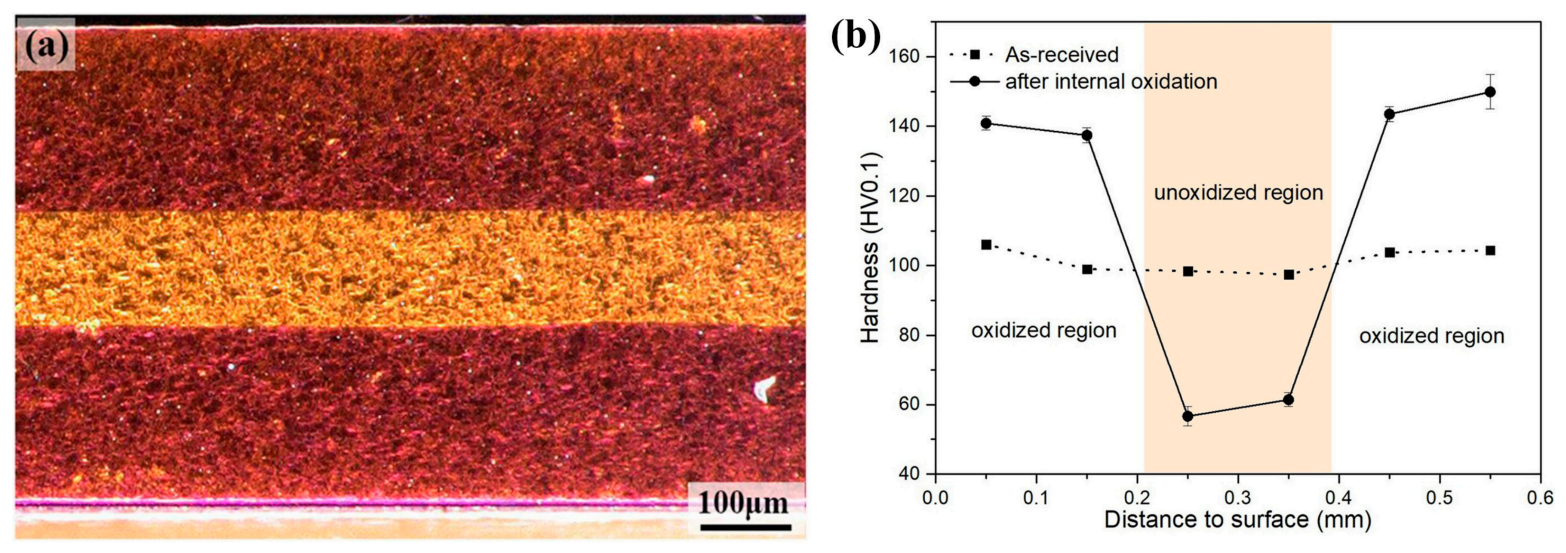
Cross-sectional microstructure and corresponding hardness profile of under-oxidized Ag-Mg-Ni alloy, showing the formation of distinct oxidized and unoxidized regions and the associated hardness gradient [7]. (**a**) Optical micrograph showing the oxidized outer layers and unoxidized core; (**b**) corresponding hardness profiles before and after internal oxidation.

According to Wagner’s parabolic relationship, extending the internal oxidation time effectively increases the depth of the internal oxidation layer and is a common method for achieving complete internal oxidation in alloys of a certain thickness [25]. For constant internal oxidation temperature and oxygen partial pressure, the internal oxidation layer depth has a linear relationship with the square root of time. Therefore, as the internal oxidation depth increases, the required oxidation time increases proportionally to the square of the depth. When material thickness is large, solute content is high, or the oxidation rate is low, incomplete oxidation may occur due to insufficient oxidation time, leaving an unoxidized region of certain depth within the alloy (Figure 7) [7]. The unoxidized region has significantly lower hardness than the oxidized region, leading to microstructural and property inhomogeneity across the cross-section.

**Figure 8:**
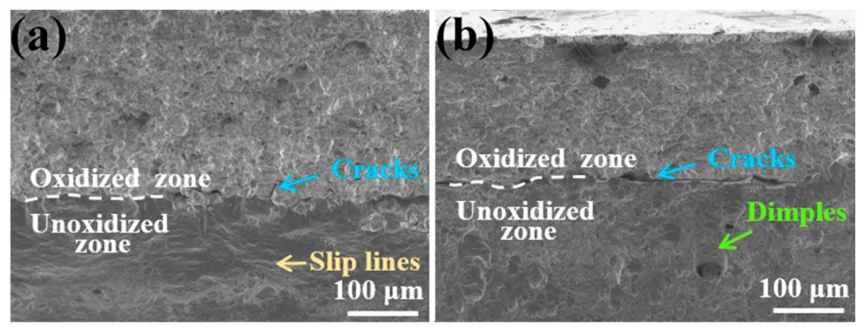
Fracture morphology of Ag-Mg-Ni alloy, illustrating interfacial crack propagation between oxidized and unoxidized zones [16]. (**a**) Ag-0.24 wt.% Mg-0.7 wt.% Ni; (**b**) Ag-0.24 wt.% Mg-2 wt.% Ni.

Liu and co-workers studied Ag-0.24 wt.% Mg-0.3 wt.% Ni alloy internally oxidized at 800 °C for 0.5 h and 8 h, obtaining incompletely and completely internally oxidized alloys, respectively [16,66]. Results showed that completely internally oxidized Ag-Mg-Ni alloy exhibited both higher strength and plasticity. In contrast, due to the presence of a softer unoxidized core, the overall load-bearing capacity of the incompletely oxidized alloy was significantly reduced, leading to a substantial decrease in strength. Additionally, the significant strength difference between the unoxidized and internally oxidized regions easily led to stress concentrations at the interface, inducing crack initiation and subsequent fracture (Figure 8) [16].

Therefore, for alloys of a certain thickness, sufficient internal oxidation time is crucial to eliminate unoxidized regions, promote complete internal oxidation, and improve microstructural and property uniformity, thereby achieving simultaneously higher strength and plasticity.

### 4.3. Oxygen Partial Pressure: The High-Pressure-Enhanced Penetration Effect

Oxygen partial pressure is another key factor controlling internal oxidation kinetics. Under low-oxygen partial pressure or insufficient oxygen supply, oxygen entering the material is easily consumed rapidly by surface solutes, reducing the oxygen flux reaching deeper regions, which slows down or even stops the advance of the oxidation front. Increasing oxygen partial pressure enhances oxygen surface activity and inward transport flux, thereby promoting the continuous advance of the oxidation front. Thus, high-oxygen partial pressure offers the possibility of achieving deeper internal oxidation layers at lower temperatures or shorter times.

**Figure 9:**
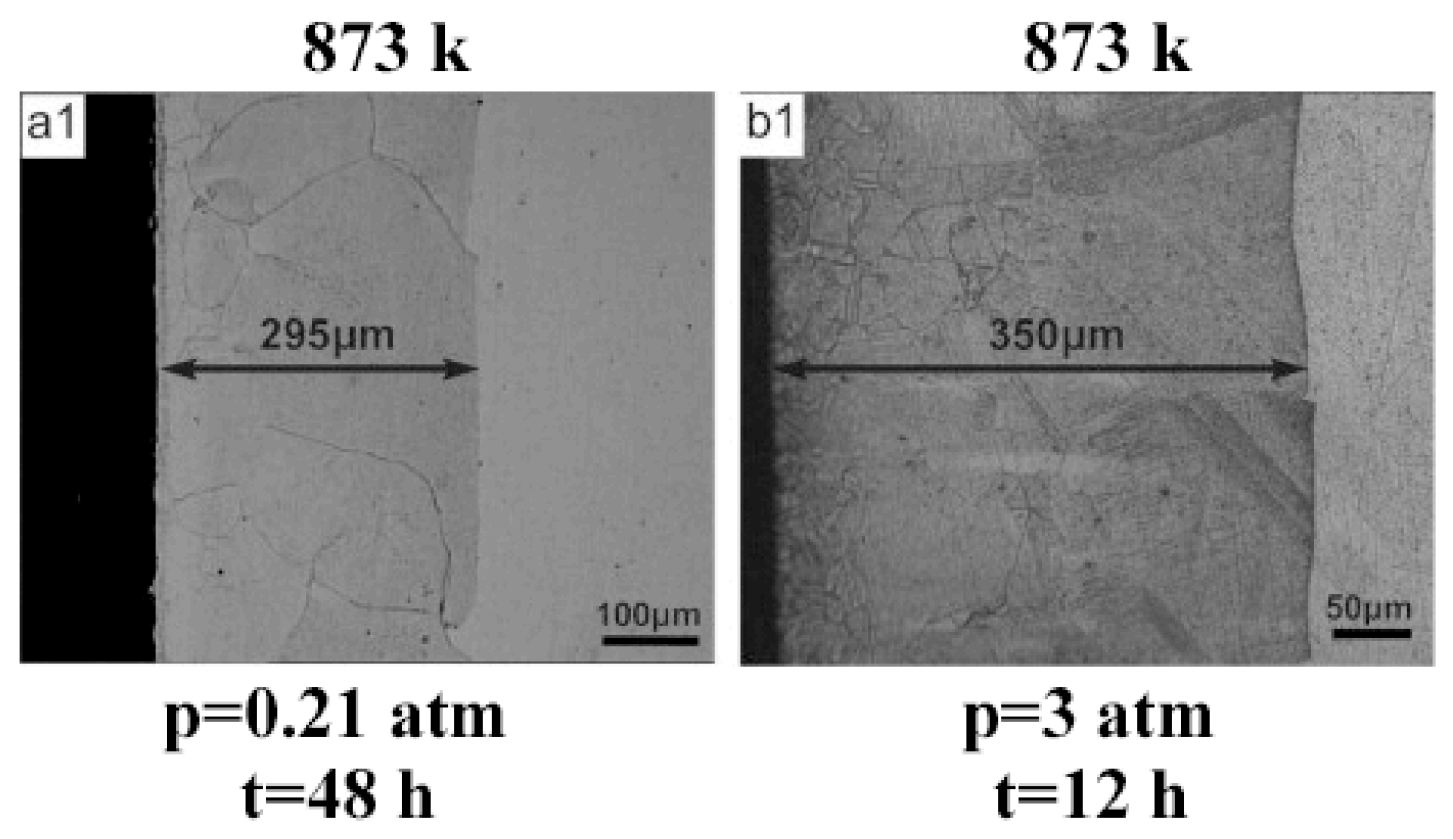
Effect of oxygen partial pressure on internal oxidation behavior in Ag-3 at.% Sn alloy at 873 K, illustrating enhanced inward oxidation due to increased oxygen supply [30]. (**a1**) 0.21 atm for 48 h; (**b1**) 3 atm for 12 h.

Liu et al. systematically investigated the effect of oxygen partial pressure on the internal oxidation behavior of Ag-based alloys [67]. Results showed that under 7.5%, 21% and 100% oxygen atmospheres, increasing oxygen partial pressure significantly increased the internal oxidation rate, achieving deeper internal oxidation layers within the same timeframe. In their study of internal oxidation of Ag-3 at.% Sn alloy at 873 K, Xu et al. found that by increasing oxygen partial pressure from 0.21 atm to 3 atm, even though the oxidation time was shortened from 48 h to 12 h, the internal oxidation depth still significantly increased (Figure 9) [30]. At 973 K, increasing oxygen partial pressure from 0.21 atm to 3 atm increased the oxidation rate constant from approximately 2.32 × 10^−8^ cm^2^/s to 8.56 × 10^−8^ cm^2^/s, an increase of about 3.7 times, significantly reducing the time required to reach an internal oxidation depth of approximately 600 μm.

Oxygen partial pressure determines the chemical potential of oxygen at the alloy surface and its equilibrium concentration in the Ag matrix. After increasing oxygen partial pressure, a higher oxygen concentration gradient can be maintained inside the material, allowing oxygen to more easily pass through the oxidized region and continue diffusing inward [68]. For Ag-Sn alloys with higher solute content, increasing oxygen partial pressure enhances the inward flux of oxygen, mitigating the limitation on deep oxygen supply caused by SnO_2_ oxide precipitation at the front. Therefore, high-oxygen partial pressure can promote internal oxidation of higher Sn content alloys.

Oxygen partial pressure also affects the particle size of oxidation products. High-oxygen partial pressure leads to a high flux of oxygen diffusing into the alloy, promoting simultaneous nucleation at more sites and increasing the number of nuclei. Simultaneously, the amount of solute available per individual nucleus decreases, leading to smaller individual particle sizes. In the Ag-Mg system, Combe et al. investigated the effect of oxygen partial pressure in the range of approximately 0.2–1 atm [69]. As oxygen partial pressure increased, the resistivity peak during internal oxidation increased, indicating that high oxygen flux increased the nucleation rate, leading to smaller but more numerous Mg-O clusters.

Therefore, for thicker materials and high-solute-content alloys, pressurized oxidation is a more ideal internal oxidation treatment method, albeit at the cost of increased equipment complexity and processing cost.

### 4.4. Third-Element Additions: Expanding the Process Window and Refining Microstructure

Introducing a third element is an effective means to overcome the depth limitations of binary alloy internal oxidation, improve the stability of oxidation front propagation and regulate the size and spatial distribution of oxides. Third elements can alter the nucleation, growth and interface characteristics of oxides, and also affect solute diffusion, grain boundary structure and recrystallization processes [70]. Due to differences in oxygen affinity, diffusion capability and oxide structure among elements, their specific mechanisms of action are highly system dependent [71].

The main problem with Ag-Sn alloys is that when Sn content increases, oxides tend to be highly concentrated at the oxidation front or in the surface layer, thereby hindering further oxygen entry. Therefore, the key to control is to inhibit the transformation of SnO_2_ from dispersed particles to continuous layers at the oxidation front, thereby achieving synergistic optimization of internal oxidation depth and oxide distribution. The introduction of elements such as Te, In, Sb and Bi can alter the sequence and spatial distribution of oxide formation [72]. Lee et al. showed that adding 0.3–0.5 at.% Te to Ag-Sn alloys prepared by induction melting and subsequent hot rolling significantly improved their internal oxidation capability at 750–800 °C [54]. Sn/Te composite oxides preferentially nucleated, increasing the number of oxide nuclei and accelerating Sn oxidation, which made the oxidation front advance faster relative to the rate of Sn enrichment, thus preventing oxide concentration at the oxidation front. After introducing approximately 0.3 at.% Te into Ag-12.2 at.% Sn alloy, complete internal oxidation of a 1.5 mm thick plate was achieved, with oxides uniformly dispersed in the Ag matrix. In the Ag-Sn-In system, In is more easily oxidized than Sn, thus preferentially forming In_2_O_3_. Studies have suggested that In_2_O_3_ can act as heterogeneous nucleation sites for SnO_2_, causing SnO_2_ to nucleate dispersedly at multiple locations rather than forming a continuous, dense SnO_2_ layer [73,74]. Elements like Sb act similarly to In, tending to preferentially form discontinuous oxides such as Sb_2_O_3_, which alters the original nucleation and growth modes of SnO_2_, disrupting the continuous growth trend of oxides, and thereby inhibiting the formation of layered oxide structures [75]. Discontinuous oxide structures help maintain continuous oxygen diffusion into the alloy, reducing the diffusion barrier at the front caused by the formation of dense oxide layers, thereby maintaining a high oxygen flux at the oxidation front and delaying the formation of dense oxide layers while promoting the continuous advance of the oxidation front. Related kinetic studies also indicate that the introduction of third elements can significantly increase the oxidation rate constant (Table 2), further confirming the role of third-element additions in regulating the diffusion–reaction process [54].

**Figure 10:**
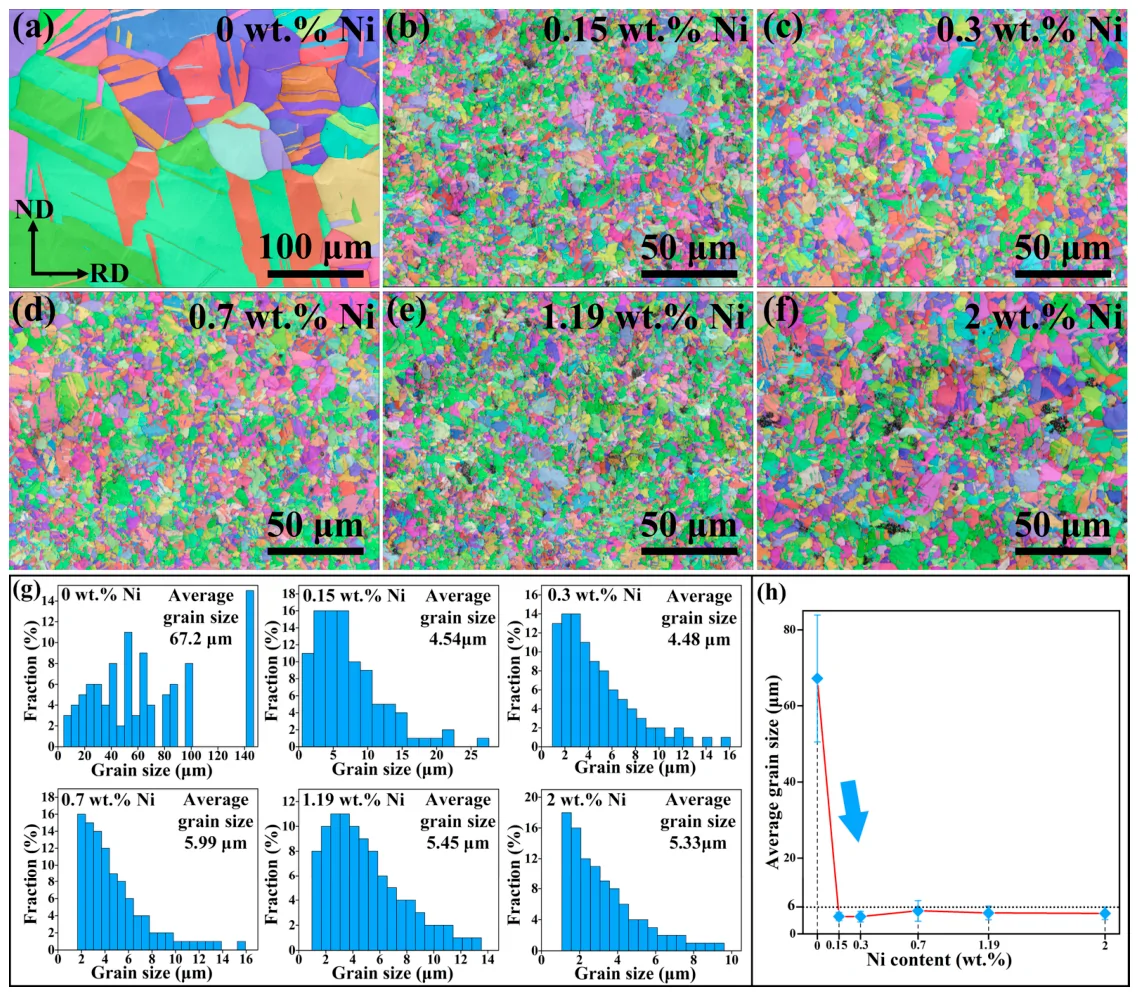
Comparison of grain structures and grain sizes of Ag-Mg and Ag-Mg-Ni alloys [66]. EBSD orientation maps of alloys containing (**a**) 0; (**b**) 0.15; (**c**) 0.3; (**d**) 0.7; (**e**) 1.19; (**f**) 2wt.% Ni; (**g**) corresponding grain size distributions; (**h**) average grain size as a function of Ni content.

**Figure 11:**
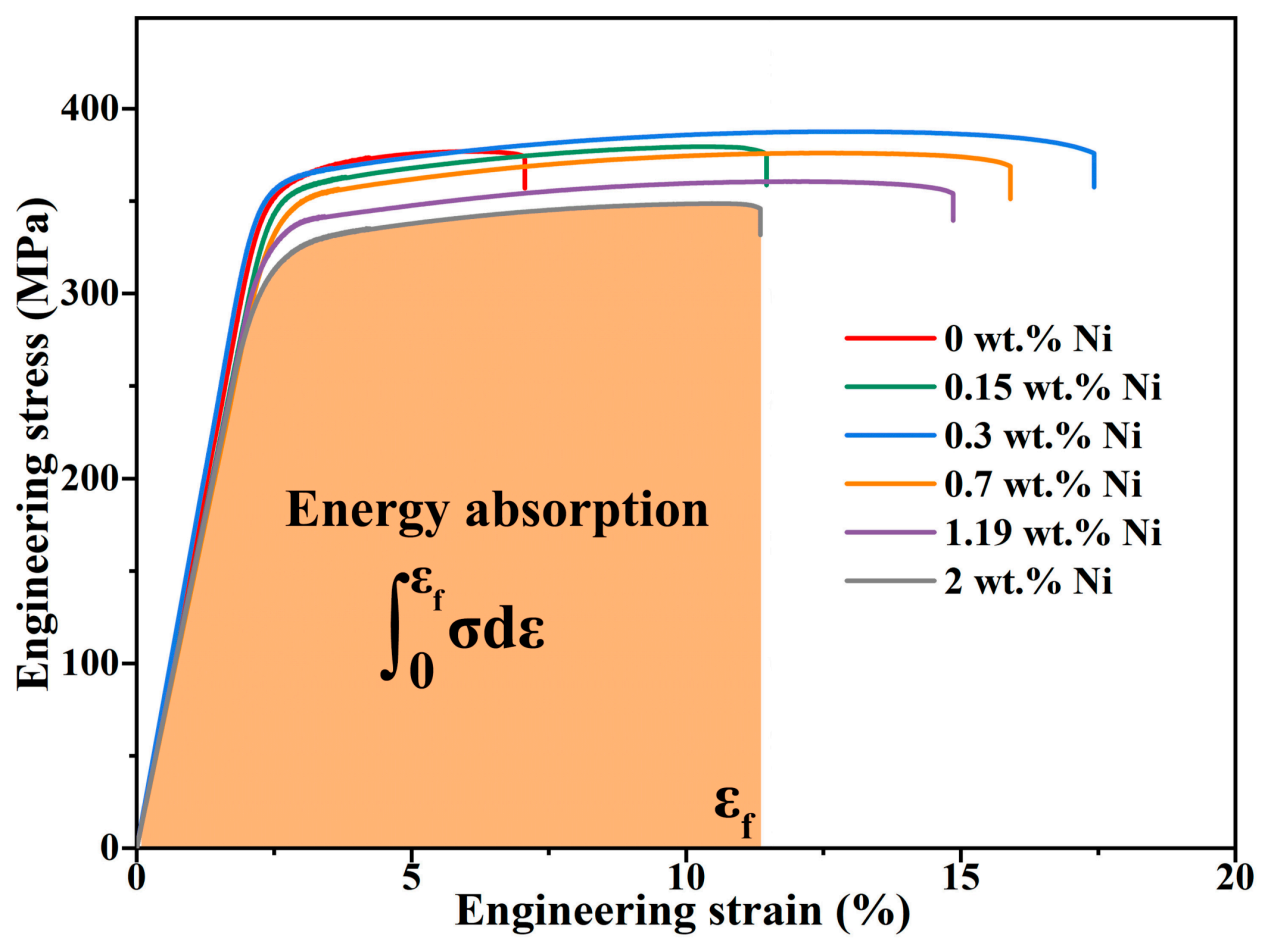
Engineering stress–strain curves for Ag-Mg and Ag-Mg-Ni alloys [66].

In the Ag-Mg system, Ag-0.24 wt.% Mg alloys with Ni additions ranging from 0 to 2.00 wt.% were prepared by induction melting and cold rolling, then internally oxidized at 800 °C for 8 h at 1 atm oxygen [66]. During oxidation, nanoscale Ni-containing particles pinned the grain boundaries and inhibited grain growth, refining the grain size from approximately 67 μm to below 6 μm (Figure 10). This refinement not only improves the uniformity of the oxide dispersion but also enhances the mechanical properties, achieving simultaneous gains in strength and ductility. Specifically, tensile strength increased to approximately 387 MPa with a fracture strain of about 17.4%. Figure 11 compares the engineering stress–strain curves of Ag-Mg-Ni alloys with different Ni contents and the binary Ag-Mg alloy [66].

Beyond strength and ductility, the tensile toughness—defined as the energy absorbed per unit volume up to fracture and calculated as the area under the engineering stress–strain curve—provides a quantitative measure of energy absorption during tensile deformation. The absorbed energy increased from approximately 21.78 MJ·m^−3^ in the Ni-free Ag-Mg alloy to 61.86 MJ·m^−3^ in the Ag-Mg-0.3 wt.% Ni alloy, a 2.8-fold improvement. This demonstrates that moderate Ni addition, through grain refinement, successfully achieves simultaneous strengthening and toughening of the internally oxidized alloy.

## 5. Mechanical-Property Relationships and Strengthening–Toughening Mechanisms

### 5.1. Dispersion-Strengthening Mechanics

Fine, dispersed oxides are the primary microstructural basis for strength enhancement in internally oxidized silver-based alloys. Oxides formed during internal oxidation can increase the material’s resistance to deformation by impeding dislocation motion, but the specific mechanism depends on the size, structure and interface relationship between the oxides and the Ag matrix [76]. For larger, harder oxide particles that cannot be directly cut by dislocations, dislocations typically need to bend between particles and continue their motion via the Orowan bypassing mechanism [77,78]. The Orowan strengthening increment can usually be expressed as [79,80]

$$\Delta \sigma_{Or}=M\frac{0.4Gb}{\pi \sqrt{1-\nu }\lambda }ln\left(\frac{2\bar{r}}{b}\right)$$

where M is the Taylor factor, G is the shear modulus of the Ag matrix, b is the Burgers vector of the dislocation, $\bar{r}$ is the average radius of the oxide particles and λ is the effective inter-particle spacing on the slip plane. This indicates that the strengthening effect of oxides is not solely dependent on particle size but is also influenced by the number, volume fraction and spatial distribution of particles. Wasserbäch et al. studied the strengthening mechanisms in internally oxidized Ag-Sn-In alloys, finding that as the volume fraction of sub-micron oxide particles increased from 0.12 to 0.20, and the inter-particle spacing decreased from 0.78 μm to 0.59 μm, the material’s yield strength increased from 142 MPa to 182 MPa [79]. The calculated Orowan strengthening contribution increased from 81 MPa to 97 MPa, indicating that increasing the volume fraction of oxide particles and reducing their inter-particle spacing can significantly enhance their impedance to dislocation motion. Therefore, for silver-based alloys where non-shearable oxides are the primary strengthening phase, the key to internal oxidation strengthening is to control the nucleation and growth of oxides through alloy composition and oxidation processes, ensuring they remain fine, dispersed and have small effective inter-particle spacing to fully exploit the Orowan strengthening effect.

However, for Ag-Mg alloys, the Mg-O clusters formed during internal oxidation typically have smaller sizes and often maintain a coherent relationship with the Ag matrix [81]. Their strengthening mechanism thus exhibits significant differences and is not solely controlled by a simple Orowan bypassing mechanism. Mahajan et al., through comparative studies and strengthening contribution calculations on internally oxidized Ag-Mg and Ag-Cd single crystals, found that CdO particles in Ag-Cd alloys were tens of nanometers in size, and their strengthening behavior conformed to the typical Orowan mechanism [82]. In contrast, the strengthening produced by Mg-O clusters smaller than 5 nm in Ag-Mg alloys was not primarily controlled by the Orowan mechanism. The internal strain fields induced by the clusters and the difference in elastic modulus between them and the Ag matrix were important sources of strengthening. Zhu et al. further revealed the structural origin of this strain field at the atomic scale, finding that internally oxidized Ag-Mg alloys formed approximately 3 nm Mg-O clusters coherent with the Ag matrix. The (200) plane spacing of Ag near the clusters increased from 0.206 nm to 0.214 nm, representing a local lattice expansion of approximately 3.9% [41]. This lattice distortion and local strain field caused by coherent clusters increase the resistance to dislocation motion, thus constituting an important strengthening mechanism for Ag-Mg alloys. Therefore, by adding a small amount of Mg to silver and utilizing internal oxidation to form fine, coherent and dispersed Mg-O clusters, significant strengthening can be achieved.

### 5.2. Hall–Petch Strengthening

Internal oxidation often requires prolonged high-temperature exposure, which is frequently accompanied by recrystallization, grain boundary migration and grain growth. Coarsened grains not only weaken the impedance of grain boundaries to dislocation motion but can also lead to microstructural and property inhomogeneity [64]. However, by controlling internal oxidation processes and third-element additions, the formation of fine oxides or nanoparticles can be promoted. These can inhibit grain coarsening by increasing recrystallization nucleation density or pinning migrating grain boundaries [16]. Therefore, in addition to the dispersion strengthening provided by the oxides themselves, grain refinement during internal oxidation can lead to Hall–Petch strengthening, where the yield strength is typically expressed as [83]

$$\sigma_{y}=\sigma_{0}+k_{y}d^{-1/2}$$

where $\sigma_{y}$ is the yield strength, $\sigma_{0}$ is the friction stress of the lattice, $k_{y}$ is the Hall–Petch coefficient and d is the average grain size. As grain size decreases, the number of grain boundaries per unit volume increases, and dislocation motion and transmission across grain boundaries are more strongly impeded, thereby increasing the material’s resistance to deformation.

Wu et al. investigated the effect of grain size on the hardness of Ag-Sn-Bi-Cu alloys [84,85]. Pre-oxidation with Ag_2_O powder improved the oxygen supply capacity and oxide nucleation rate within the powder, resulting in finer, more uniform oxides and enhanced pinning of Ag grain boundaries. This inhibited grain growth during 900 °C treatment. Compared to alloys oxidized in air, the average grain size of the Ag matrix decreased from 5.79 μm to 2.69 μm, and the material hardness increased from 60 HV to 70 HV. Zhu et al. studied grain evolution during internal oxidation of Ag-Mg and Ag-Mg-Ni alloys [66]. Results showed that nanoscale-coherent Mg-O clusters formed during internal oxidation in Ag-Mg alloys were mainly distributed within grain interiors and, although producing significant cluster strengthening, had limited ability to inhibit grain growth during high-temperature internal oxidation. After introducing Ni, which has negligible solid solubility in the Ag matrix, Ni particles pinned grain boundaries during internal oxidation, preventing grain growth and enabling fine, uniform grain microstructures, thereby achieving strength–ductility synergy.

### 5.3. Grain-Refinement Toughening

Throughout this review, “toughness” refers specifically to tensile toughness, defined as the energy absorbed per unit volume up to fracture, obtained as the area under the engineering stress–strain curve and expressed in MJ·m^−3^. This is a measure of the work of deformation prior to failure. It is distinct from fracture toughness, which quantifies the resistance of a material to crack propagation and is characterized by parameters such as *K*_IC_. Because the latter requires dedicated fracture-mechanics testing that has not been reported for internally oxidized Ag-based alloys, the present discussion is restricted to tensile toughness.

The dispersed oxides and nanoclusters formed by internal oxidation effectively impede dislocation motion and strengthen Ag-based alloys, but they may simultaneously restrict plastic deformation and reduce ductility. Toughening of internally oxidized Ag-based alloys therefore requires not only retaining effective matrix strengthening but also improving the deformation and fracture behavior through microstructural control. Grain refinement offers an important route to this end: a fine, uniform grain structure improves deformation compatibility among neighboring grains, mitigates local strain concentration and reduces the tendency for premature cracking at coarse grains and their boundaries. Grain refinement thus not only strengthens the alloy via the Hall–Petch mechanism but also improves its deformability and fracture behavior.

Zhu et al. investigated this strategy in fully internally oxidized Ag-Mg and Ag-Mg-Ni alloys [66]. Ag-0.24 wt.% Mg alloys with Ni additions of 0–2.00 wt.% were prepared by induction melting and cold rolling, then internally oxidized at 800 °C for 8 h at 1 atm oxygen. During oxidation, nanoscale Ni-containing particles pinned the grain boundaries and inhibited grain growth, refining the grain size from approximately 67 μm to below 6 μm (Figure 10). In both alloys, dispersed Mg-O nanoclusters formed and provided substantial matrix strengthening; the Ni addition did not alter this strengthening contribution. The two alloys differed, however, in their deformation and fracture behavior. The coarse-grained Ag-Mg alloy was prone to intergranular cracking, which limited its plastic deformability. In contrast, the fine-grained Ag-Mg-Ni alloy showed markedly fewer intergranular cracks on the fracture surface and a more uniform dimpled morphology, indicating a shift toward a more ductile fracture mode. These fractographic observations are qualitative: they indicate a change in fracture mode but do not constitute a quantitative measure of tensile toughness.

Figure 11 compares the engineering stress–strain curves of Ag-Mg-Ni alloys with different Ni contents and the binary Ag-Mg alloy [66]. With 0.3 wt.% Ni addition, the tensile strength increased to approximately 387 MPa and the fracture strain reached about 17.4%, both improving simultaneously relative to the Ni-free alloy. Beyond tensile strength and fracture strain, tensile toughness provides an additional measure of the energy absorbed per unit volume up to fracture, obtained by integrating the engineering stress–strain curve. The absorbed energy increased from approximately 21.78 MJ·m^−3^ in the Ni-free Ag-Mg alloy to 61.86 MJ·m^−3^ in the Ag-Mg-0.3 wt.% Ni alloy, a 2.8-fold improvement. These results demonstrate that, while retaining the strengthening contribution of the Mg-O nanoclusters, grain refinement through Ni addition improves the deformation and fracture behavior of the alloy and nearly triples its tensile toughness. This confirms that grain refinement enables a synergistic enhancement of strength and tensile toughness in internally oxidized Ag-based alloys, rather than the strength–ductility trade-off typically associated with dispersion strengthening alone. Whether the same microstructural strategy also improves fracture toughness, as characterized by *K*_IC_, remains an open question and is identified here as a direction for future work.

### 5.4. Microstructural Limitations: Internal Oxide Band and Under-Oxidized Cores

The effectiveness of internal oxidation strengthening rests on the fine, dispersed and uniform distribution of oxides. When this ideal microstructural state is disrupted, its strengthening advantages are significantly diminished. When oxides locally aggregate to form continuous or semi-continuous internal oxide bands, the dispersion strengthening effect diminishes, and coarse oxides and their interfaces with the Ag matrix easily generate stress concentrations, making them preferential sites for crack initiation and propagation, ultimately leading to decreased plasticity and fracture resistance [6,41]. On the other hand, when incomplete oxidation occurs, the coexistence of strengthened internal oxidation zones and relatively soft unoxidized zones creates significant microstructural and property gradients within the material, reducing overall load-bearing capacity due to deformation incompatibility. Existing studies on internally oxidized Ag-based alloys indicate that brittle fracture caused by internal oxide bands and low strength resulting from incomplete internal oxidation are the two typical microstructural problems limiting the effectiveness of internal oxidation in silver-based alloys. Therefore, suppressing the formation of internal oxide bands and promoting complete internal oxidation of the alloy are key pathways to achieving strengthening and toughening of silver-based alloys.

Table 3 summarizes the reported properties and key microstructural characteristics of representative internally oxidized Ag-based alloys, providing a quantitative basis for comparing the effectiveness of the microstructural strategies outlined above.

## 6. Summary and Outlook

### 6.1. Summary

Internal oxidation of silver-based alloys is governed by a narrow window of thermodynamic and kinetic conditions, within which the microstructure—and hence the strength–ductility balance—can be tuned by processing and by third-element additions. This review has traced that chain from the governing criteria, through the microstructural evolution of the Ag-Mg and Ag-Sn systems, to the strengthening and toughening mechanisms involved. The principal conclusions are as follows:

(1) Thermodynamic and kinetic selectivity are jointly necessary. Selective internal oxidation requires both that the solute oxide be far more stable than any oxide of the matrix and that $D_{O}C_{O}^{S}\gg D_{B}C_{B}^{0}$, where $D_{O}$ and $D_{B}$ are the diffusivities of oxygen and of the solute, $C_{O}^{S}$ is the surface solubility of oxygen, and $C_{B}^{0}$ is the bulk solute concentration. Silver satisfies both criteria exceptionally well: Ag_2_O decomposes above approximately 300 °C, and oxygen diffuses through solid Ag about four orders of magnitude faster than Mg or Sn. Ag-based systems are therefore a well-established model system for Wagner’s diffusion-controlled internal oxidation model.

(2) Two characteristic microstructural defects bound the process window. At high solute contents, solute enrichment ahead of a decelerating oxidation front drives the transition from dispersed oxides to continuous or semi-continuous bands, which act both as crack-initiation sites and as barriers to further oxygen ingress. At large section thicknesses, parabolic kinetics dictate that an unoxidized core will remain unless the oxidation time is long enough; the resulting strength and hardness gradient promotes interfacial fracture.

(3) In Ag-Mg, coherency-strain hardening dominates at low Mg contents, whereas banding causes embrittlement at high contents. Below approximately 2 at.% Mg, internal oxidation produces Mg-O clusters of 3 nm that remain coherent with the Ag lattice and impose a local lattice expansion of 3.9%, markedly increasing the resistance to dislocation glide. At Mg contents of approximately 2 at.% and above, MgO band formation triggers brittle fracture. The precise threshold is process-dependent, with reported values between 2 and 3 at.%; this spread reflects differences in temperature, time and atmosphere and merits systematic study.

(4) In Ag-Sn, Orowan bypassing dominates, but banding becomes acute above ~5 at.% Sn. Quantitative analysis of Ag-Sn-In alloys confirms that the yield strength scales with the inverse of the Orowan interparticle spacing, and that third-element additions (Te, In, Sb, Bi), which nucleate discontinuous secondary oxides, can raise the kinetically tolerable Sn content by suppressing SnO_2_ band formation.

(5) Third-element additions to Ag-Mg, notably Ni, exploit grain-boundary pinning. Grain refinement from ~67 μm to below 6 μm yields a tensile strength of ~387 MPa, a fracture strain of ~17.4% and a tensile toughness that is approximately 2.8 times that of the Ni-free alloy, demonstrating the synergy between Mg-O nanocluster strengthening and grain-refinement toughening.

### 6.2. Outstanding Questions and Future Directions

Despite the progress summarized above, several scientifically important and technologically consequential questions remain open.

(1) Atomistic origin of the band-formation threshold. The critical solute content separating dispersed-cluster from banded-oxide microstructures in Ag-Mg varies between 2 and 3 at.% across the literature, with no consensus on the governing parameter. Atom-probe tomography (APT) combined with in situ transmission electron microscopy heating experiments could resolve whether this reflects a true compositional transition or sensitivity to grain-boundary diffusion, local strain and nucleation kinetics.

(2) Real-time visualization of the oxidation front. The dynamics of oxidation-front advance, solute enrichment, and oxide nucleation are inferred entirely from post-mortem cross-sections. In situ environmental TEM or synchrotron X-ray tomography during controlled oxidation would directly capture the moment of band initiation and allow Wagner’s kinetic assumptions to be tested against actual front morphologies.

(3) Quantitative multiscale modeling. The Wagner parabolic model captures the global kinetics but cannot predict oxide morphology, size distribution or band formation. Phase-field models incorporating oxygen and solute diffusion fields, oxide nucleation and elastic misfit are emerging but have yet to be validated against the full range of Ag-Mg and Ag-Sn experimental data. Machine-learning interatomic potentials trained on DFT databases for the Ag-Mg-O and Ag-Sn-O systems could accelerate this effort and enable direct computation of coherency energies and dislocation–cluster interaction forces.

(4) Third-element design beyond empirical screening. The roles of Te, In, Sb, Bi and Ni have been established primarily through trial and error. A thermodynamic–kinetic framework combining calculated oxide stability (CALPHAD), diffusivity databases and precipitation simulations could guide rational selection of new additives that suppress banding and refine grain size without relying on costly empirical iteration.

(5) Novel processing routes. Powder metallurgy and additive manufacturing (selective laser melting, directed energy deposition) of Ag-based alloys raise new questions about how rapid thermal cycling and non-equilibrium solidification affect the internal oxidation response of pre-alloyed or reactively processed feedstocks. The feasibility of in situ internal oxidation during sintering or post-build heat treatment represents an unexplored manufacturing opportunity.

(6) Service-condition microstructure stability. High-reliability electrical contacts experience repeated arc discharges, localized melting and thermal cycling. The stability of Mg-O clusters and SnO_2_ dispersions under these conditions—including coarsening kinetics, re-dissolution of solute and oxide-matrix interface evolution—has received far less attention than the as-processed microstructure and deserves systematic characterization.

## Figures and Tables

**Table 1:** Comparison of properties and applications of silver-based electrical contact materials.

Material Type	Typical Systems	Key Properties	Typical Applications
Silver–magnesium [6,7]	Ag-Mg/Ag-Mg-Ni	High strength and hardness after internal oxidation; ductility depends on alloy composition and microstructure	Spring-type (reed-like) contact components
Silver–tin oxide [8,9]	Ag-SnO_2_	Environmentally friendly alternative; good arc resistance and anti-welding capability	Medium- and high-voltage electrical devices, relays, switches
Silver–zinc oxide [10,11]	Ag-ZnO	Good electrical contact performance and wear resistance; anti-welding performance depends on composition	Medium- and low-load contacts
Silver–aluminum [12,13]	Ag-Al/Ag-Al_2_O_3_	High hardness and wear resistance; oxidation may increase contact resistance	Electrical contact components and sliding contacts
Silver–refractory metals [14]	Ag-W	High melting point; excellent arc erosion resistance	High-voltage and high-current switching devices
Silver–graphite [15]	Ag-C	Self-lubricating; excellent anti-adhesion behavior	Sliding contacts

**Table 2:** Approximate internal oxidation rates of Ag-Sn-based alloys at 800 °C.

Alloys	Additional Element	Oxidation Rate (μm·h^−1/2^)
Ag-3Sn	/	~265
Ag-3Sn	Te	~395
Ag-5Sn	/	~35
Ag-7Sn	Bi	~120
Ag-10Sn	Te	~270

Oxidation rates were estimated from Ref. [54] and are used for comparison only.

**Table 3:** Summary of reported properties of internally oxidized Ag-based alloys.

Alloy	Hardness (HV)	Strength (MPa)	Elongation (%)	Key Feature	Ref.
Ag-1.2 at.% Mg	/	YS~383	/	Coherent Mg-O clusters	[43]
Ag-1 at.% Mg	178	UTS~269	3.9	Nanocluster zone, no bands	[41]
Ag-7 at.% Mg	224	UTS~138	0.4	Band formation	[41]
Ag-0.24 wt.% Mg-0.30 wt.% Ni	143	UTS~387	17.4	Grain refinement to <6 μm	[66]
Ag-Sn-Bi-Cu	70	/	/	Fine oxides	[85]
Ag-Sn-In	89–95	YS~142-182	15–33	Orowan regime	[22,79]

## Data Availability

No new data were created or analyzed in this study. Data sharing is not applicable to this article.
